# Vascular Decoupling in Septic Shock: The Combined Role of Autonomic Nervous System, Arterial Stiffness, and Peripheral Vascular Tone

**DOI:** 10.3389/fphys.2020.00594

**Published:** 2020-07-07

**Authors:** Marta Carrara, Antoine Herpain, Giuseppe Baselli, Manuela Ferrario

**Affiliations:** ^1^Department of Electronics, Information and Bioengineering, Politecnico di Milano, Milan, Italy; ^2^Experimental Laboratory of Intensive Care, Erasme Hospital, Université Libre de Bruxelles, Brussels, Belgium; ^3^Department of Intensive Care, Erasme Hospital, Université Libre de Bruxelles, Brussels, Belgium

**Keywords:** sepsis, autonomic cardiovascular regulation, vascular tone, pulse pressure, capillary leakage, arterial compliance

## Abstract

**Background:**

Acute inflammation and sepsis are known to induce changes in vascular properties, leading to increased arterial stiffness; at the same time, the autonomic nervous system (ANS) also affects vascular properties by modulating the arterial smooth muscle tone, and it is widely reported that sepsis and septic shock severely impair ANS activity. Currently, clinical guidelines are mainly concerned to resuscitate septic shock patients from hypotension, hypovolemia, and hypoperfusion; however, if the current resuscitation maneuvers have a beneficial effect also on vascular properties and autonomic functionality is still unclear. The objective of this work is to assess the effects of standard resuscitation at vascular level and to verify if there is any association between alterations in vascular properties and ANS activity.

**Methods:**

Six pigs underwent a protocol of polymicrobial septic shock and resuscitation (fluids and noradrenaline). The arterial blood pressure (ABP) waveform was recorded in the central aorta and in the peripheral radial and femoral artery. The characteristic arterial time constant was computed at the three arterial sites based on the two-element Windkessel model, to characterize the overall arterial vascular tree. Moreover, independent estimates of total arterial compliance (AC) and total peripheral resistance (TPR) were performed. Baroreflex sensitivity (BRS), low frequency (LF, 0.04–0.15 Hz) spectral power of diastolic blood pressure, and indices of heart rate variability (HRV) were computed to assess ANS functionality.

**Results:**

Septic shock induced a severe vascular disarray, decoupling the usual pressure wave propagation from central to peripheral sites; this phenomenon appeared as an inversion of the physiological pulse pressure (PP) amplification, with a higher PP in the central aorta than in the peripheral arteries. The time constant was decreased, together with AC and TPR. ANS dysfunction was described by a reduced BRS, decreased LF power, and suppressed HRV. This compromised condition was not resolved by administration of fluids and noradrenaline. Thus, a persistent vascular and autonomic dysfunction were reported also in the resuscitated animals, and they were found to be significantly correlated.

**Conclusion:**

Measures of vascular function and ANS activity could add information to standard hemodynamic and clinical markers, and the current resuscitation strategies could benefit from the adjunction of these additional functional indices.

## Introduction

Acute inflammation and sepsis are known to impair endothelial functions, leading to an imbalance of vasodilatory and vasoconstrictive mechanisms, which may lead to multi organ failure (Ince et al., 2016). Much research has focused on the analysis of the mechanisms underlying vasodilation of resistance vessels, but a major role is also played by mechanical properties of compliant arteries, especially the aorta. Elastic arteries are able to accommodate the ejected stroke volume and they allow to ensure the optimal flow conditions in the periphery, including during diastole. A reduction in the elastic properties (i.e., arterial stiffness) of the large arteries, and in particular of the aorta, leads to increased left ventricular afterload (increased myocardial oxygen demand), reduced coronary perfusion (decreased myocardial oxygen delivery), and mechanical fatigue of the arterial walls (Vlachopoulos et al., 2005).

Sepsis and septic shock were proved to significantly increase the stiffness of the large arteries. For example, LPS injection in rabbits has been shown to induce a dysfunction of the endothelium-mediated vascular relaxation in the aorta (Leclerc et al., 2000), and additional experimental observations in endotoxin shock swine model confirmed an increased stiffness of the central aorta and, on the opposite, an increase in compliance of the peripheral compartment generated by septic shock (Hatib et al., 2011). Finally, a recent clinical observational study on arterial elastic properties has shown an increase in aortic stiffness, with respect to the general population, in a cohort of septic shock patients when pulse wave velocity (PWV, an indirect measure of arterial stiffness in large arteries) was measured at the time of admission (Kazune et al., 2019).

At the same time, the sympathetic autonomic nervous system (ANS) physiologically contributes, mainly through the release of noradrenaline, to the modulation of arterial smooth muscle tone; thereby it interacts with local endothelial mechanisms and it may affect the arterial mechanical properties. A relationship between muscle sympathetic nerve activity—an invasive measure of the peripheral ANS—and PWV was observed in healthy individuals, regardless of any other cofounding factors (Swierblewska et al., 2010), and an association between alterations in vascular properties (arterial stiffness) and dysfunction of the ANS has been widely documented in several chronic pathologies, including diabetic patients (Liatis et al., 2011; Secrest et al., 2011; Theilade et al., 2013), hypertensive patients (Carthy, 2014), or heart failure patients (Millar et al., 2019).

Current clinical guidelines are mainly concerned to recover septic shock patients from hypotension and hypovolemia and the mean value of arterial pressure is one of the key therapy targets (Rhodes et al., 2017); however, no further recommendation is addressed about the more subtle changes occurring in the arterial pressure waveform, which can give insight on peripheral vascular tone, backward pressure waves, and large arteries compliance, and suffer from significant modifications. Interestingly, a recent clinical observational study pointed out the effects of noradrenaline administration in septic shock patients, highlighting that high doses of noradrenaline increase arterial characteristic impedance, pulse wave velocity, and reflection phenomena, while reducing aortic compliance (Monge García et al., 2018).

Given these premises, the main objectives of this study are (1) to measure the vascular system properties, at central and peripheral levels, in an experimental model of polymicrobial septic shock with standard resuscitation, i.e., fluids and vasopressors administration; (2) to assess the effects of these therapies at vascular level and to extend the previous observations made on endotoxin shock models (Hatib et al., 2011). Finally, we want to verify if these alterations of the vascular system properties in septic shock are accompanied by altered ANS regulatory mechanisms of arterial pressure.

## Materials and Methods

### Study Design and Experimental Procedure

This experimental study has been performed in the Experimental Laboratory of Intensive Care (LA1230336), at the Université Libre de Bruxelles, on a large animal model of septic shock induced on young adult swine by a fecal peritonitis. The local animal ethics committee (Comité Ethique du Bien-Être Animal) approved the present study (protocol 641 N) and we followed the EU Directive 2010/63/EU for animal experiments, the ARRIVE guidelines for animal research, and the MQTiPSS recommendations for sepsis translational research (Osuchowski et al. in Shock, 2018).

Six pigs of both sex (age 4–6 months, weight 43.8 ± 3.9 kg expressed as mean ± standard deviation) were obtained from a local farm (BE 400108–48). Animals were fasted for 18 h prior to the experiment with free access to water. Details on the instrumentation and experiment preparation can be found in the Supplemental Material of a previous article (Carrara et al., 2019). Briefly, the instrumentation was performed with a closed chest and a minimally invasive approach, having all the catheters or vascular sheets introduced only by a percutaneous approach under echographic guidance; after which the animals were allowed to rest for 2 h before the first baseline hemodynamic measurements (baseline, T1). Sepsis was induced by the intraperitoneal instillation, via the two abdominal drains, of 3 g/kg of autologous feces collected in the cage, filtered and diluted in 300 ml of glucose 5%. Since the sepsis onset, a balanced crystalloid perfusion (Plasmalyte^®^, Baxter, Belgium) served as the only maintenance perfusion, at a very low rate (1 ml/kg/h) and, as soon as the animal reached a hypotensive state of mean arterial pressure (MAP) < 50 mmHg, it was modestly increased to keep this MAP between 45 and 50 mmHg for one last hour, in order to consolidate the peripheral hypoperfusion and the multiple organ failure onset (corresponding in practice to less than 400 ml in every animal during this last hour before massive fluid resuscitation). At the end of this period, a second time point T2 was defined as reference for non-resuscitated septic shock condition. Immediately after a series of hemodynamic measurements recording, a full fluid resuscitation was initiated with both the same rate of balanced crystalloid perfusion (Plasmalyte^®^, Baxter, Belgium) and colloid perfusion (Geloplasma, Fresenius Kabi, France), aiming at reaching an arterial pulse pressure variation (PPV) < 12%. After 120 min of hemodynamic stabilization, defined by the absence of further increase in cardiac output and a stable PPV, additional hemodynamic measurements were recorded again (T3, end of first resuscitation). Finally, a continuous infusion of norepinephrine at a fixed dose of 0.3 μg/kg/min was administered and after 1 h, the last series of hemodynamic measurements were recorded again (T4, end of full resuscitation). Animal were then euthanized with a potassium chloride injection and an overdose of thiopental.

Two out of six animals were immediately fully resuscitated with vasopressors because of the severity of the distributive shock, therefore the measurements at time point T3 are missing. For this reason, in the following, we refer only to baseline (T1), shock (T2), and to fully resuscitation (T4) period. We want to underline that the early introduction of vasopressor in two pigs allowed us to keep the animal cohort more homogeneous, by avoiding some animals to suffer from prolonged and irreversible organs hypoperfusion and hypoxia, while others did not. In this way, we ended up at T4 with all the animals receiving exactly the same preload optimization and the same dose of noradrenaline.

### Hemodynamic Data Acquisition and Preprocessing

Arterial blood pressure (ABP) was continuously recorded during the experiment at three different sites: in the ascending aorta (trough an internal carotid artery puncture), in the common femoral artery and in the radial artery. Aortic and femoral arterial pressures were measured using high-fidelity pressure transducers on solid tip catheters (respectively: 5F pressure catheter, Transonic System Europe, Netherlands and SPR-350S Mikro-Tip^®^, Millar, United States). Radial arterial pressure was measured using a fluid filled catheter (3F Leader Cath, Vygon, France) and an external pressure transducer (TrueWave^®^, Edwards, Belgium); potential damping or resonance artifacts were excluded by careful cares and regular fast flush tests (so-called Gardner’s test). Each arterial pressure signal was exported to an A/D recording station (Notocord Hem, Notocord, France), similarly to the surface ECG signal, with a high temporal resolution (100–500 Hz).

At each time point stationary segments of about 15-min length were selected. Time series of systolic (SAP), diastolic (DAP), mean (MAP) pressures, and pulse pressures (PPs), computed as the difference between SAP and DAP within the same ABP pulse, were obtained from ABP waveform using standard algorithms (Zong et al., 2003; Sun et al., 2004). The time series of heart period (HP) was obtained from the aortic ABP waveform by computing the time difference between two consecutive onsets of ABP beats and considered as a surrogate of the RR-intervals time series. An adaptive filter was then applied to the time series in order to remove outliers and irregularities (Wessel et al., 2000) and each time series was finally resampled at 2 Hz by means of a zero-order hold technique. The pre-processed time series were then subdivided into 3-min 50% overlapping windows and each window was detrended using a high-order polynomial function. In order to guarantee stationarity, the Kwiatkowski–Phillips–Schmidt–Shin (KPSS) statistical test was performed. Except for the time constant τ described in the following, the indices considered for successive statistical comparisons are the average of the ones obtained from each 3-min window.

Continuous cardiac output CO (l min^–1^) monitoring was performed using a pulmonary artery catheter (CCO; Edwards LifeSciences, Irvine, CA, United States), from which were computed the continuous stroke volume SV (ml).

### The Windkessel Time Constant τ

**FIGURE 1:**
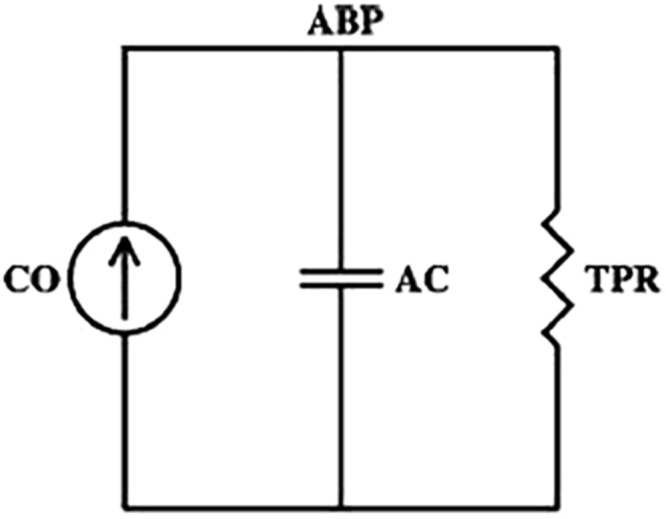
The two-element Windkessel model of arterial tree. CO, cardiac output; ABP, arterial blood pressure; TPR, total peripheral resistance; AC, arterial compliance. According to the model, the time constant τ can be computed as the product of TPR and AC (τ = TPR*AC).

According to the two-element Windkessel model reported in Figure 1, the time constant τ of the arterial tree can be computed by the following equation:

τ=T⁢P⁢R*A⁢C

where TPR is the total peripheral resistance and AC is the total arterial compliance (Westerhof et al., 2009). This relationship is true on a beat-to-beat basis if we consider the ABP waveform measured centrally where the cumulative effects of wave reflections are attenuated (Bourgeois et al., 1974); in this case, ABP should decay like a pure exponential during each diastolic interval with a time constant τ. However, this relationship is also consistent if we consider a time scale sufficiently long such that the wavelengths of the propagating waves are much larger than the dimension of the arterial tree. At such time scales, the arterial tree acts as a single blood reservoir, and the Windkessel model is therefore valid. So, for example, if pulsatile activity abruptly ceased, then peripheral ABP may eventually decay like a pure exponential as soon as the faster wave reflections died out (Mukkamala et al., 2006).

Based on this concept, we computed the time constant τ on long time intervals (6-min windows) of the measured ABP waveforms by adopting the method proposed by Mukkamala et al. (2006). In particular, the technique is specifically implemented in three mathematical steps (Mukkamala et al., 2006).

First, a cardiac contractions signal *x*(*t*) is created, which consists in an impulse train where each impulse is placed at the time instant of the onset of ABP upstroke and has an area equal to the ensuing PP.

Then, the relationship between the cardiac contractions signal *x*(*t*) and the ABP waveform named *y*(*t*) is characterized by estimating an impulse response function *h*(*t*), which is found by minimizing in the least squares sense the convolution with *x*(*t*) in order to best fit *y*(*t*). By mathematical definition, the estimated *h*(*t*) represents the ABP response to a single cardiac contraction (normalized approximately by the average PP).

**FIGURE 2:**
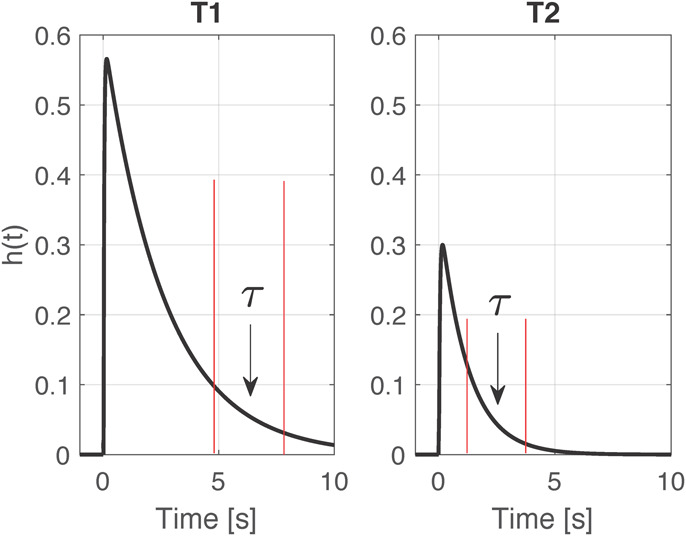
Example of two impulse responses *h*(*t*) computed at time point T1 and T2 for one pig. Highlighted in red the time windows selected for the computation of the time constant τ.

Next, the Windkessel time constant τ is determined over a selected interval after the time of its maximum value based on exponential fitting. This optimization problem is solved via linear least squares estimation by log transforming *h*(*t*). The time interval for the determination of the time constant τ is updated at each time point based on the exponential decay of *h*(*t*). An example of the obtained impulse responses at two different time points is reported in Figure 2; at T2, the faster response implies an earlier time window, as highlighted in red. The time constant τ was determined on each 6-min 50% overlapping window, then averaged for the successive statistical analysis.

The total arterial compliance AC was independently estimated with further two different methods: (i) as the ratio between continuous SV and aortic PP; (ii) as proposed in Liu et al. (1986), i.e., by using the following equation:

$$A\,C=\frac{S\,V}{K\,\left(P_{s}^{*}-P_{d}\right)}$$

where *K = (A_*s*_+A_*d*_)/A_*d*_* represents the ratio between the total area under aortic pressure and the diastolic area, i.e., the area between the dicrotic notch and the end of diastole; $P_{s}^{*}$ is the aortic pressure at the occurrence time of the dicrotic notch, and *P*_*d*_ is the aortic pressure at the end of diastole.

The total peripheral resistance TPR was computed as TPR = (MAP-RAP)/CO, according to the Windkessel model (Figure 1), where the value of right atrial pressure (RAP) is assumed to be zero, as much lower than arterial pressure and, thus, negligible. We want to underline that in shock condition, although MAP is decreased, also the value of RAP is highly reduced, and the assumption can be considered still valid. During resuscitation, the raised CO to values almost doubled compared to baseline can be considered the major driver for the decreased TPR.

### Cardiac Baroreflex Sensitivity (BRS) Analysis

The cardiac baroreflex, i.e., the ANS control of oscillations in the RR-intervals or HP induced by oscillations in SAP, was estimated via the bivariate model method on the aortic ABP. Details of the method can be found in Barbieri et al. (2001). The parameters of interest are the feedback gain (FB), which quantifies the autonomic-mediated cardiac baroreflex, and the feedforward gain (FF) or runoff effect, which explains the oscillations in blood pressure generated by oscillations in HR, due to the mechanical coupling between the two systems. Granger causality from SAP to HP and vice versa was verified before computation of the gains (Granger, 1963). The order of the model was optimized based on the Akaike information criterion, ranging from 5 to 15.

### Heart Rate Variability (HRV) Analysis and Spectral Analysis

We assessed the heart rate variability (HRV), as an indicator of the ANS control on HR, by computing the root mean square of successive differences between adjacent HP (RMSSD), the standard deviation of successive differences between adjacent HP (SDSD), and the standard deviation of the overall HP time series (SD) (Task Force of the European Society of Cardiology and the North American Society of Pacing and Electrophysiology, 1996). The analyses were performed on HP time series extracted from the aortic ABP signal.

Spectral indices obtained from power spectra of DAP included the normalized low frequency power (LFu), which represents the relative power in the low frequency (LF, 0.04–0.15 Hz) band and it is computed as the LF power divided by the total power without the very LF (VLF) component (0–004 Hz). LF oscillations in DAP values are meant to be mainly associated to the sympathetic ANS control of peripheral resistance.

### Clinical Data

Clinical variables collected at each time point were urine output UO (ml), lactate (mmol l^–1^), and mixed venous oxygen saturation SvO_2_ (%).

### Statistical Analysis

Friedman test was performed to detect differences among the three time points across multiple test attempts. In case of significant Friedman test *p*-value, i.e., <0.05, we used then the Wilcoxon signed-rank test to assess significant changes among time points within the septic shock group of animals. The values of blood pressure components (SAP, DAP, MAP, PP) were compared at each time point among the different sites (radial and femoral) taking the aorta as a reference by means of Mann–Whitney *U*-test. Parameters values are reported as median (25th, 75th percentile).

In order to test for significant associations between changes in vascular characteristics and ANS activity, correlations analyses were performed by using the Spearman correlation test.

Significance was considered with a *p*-value < 0.05.

## Results

**FIGURE 3:**
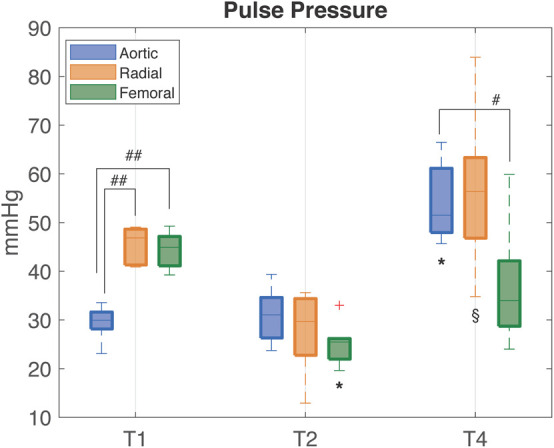
Distribution (median [25th, 75th percentile]) of PP values computed from aortic (blue), radial (orange), and femoral (green) ABP waveforms at each time point of the experiment. T1, baseline; T2, after development of septic shock; T4, after resuscitation with fluids and noradrenaline. Wilcoxon signed test: ^∗^*p*-value < 0.05 with respect to T1, ^§^ *p*-value < 0.05 with respect to T2 (Friedman test *p*-value < 0.05). Mann–Whitney *U*-test: ^#^*p*-value < 0.05 between the specified variables.^##^*p*-value < 0.01.

Table 1 provides the median values (25th, 75th percentile) of the hemodynamic parameters, the clinical variables, the estimated total arterial compliance AC, and the total peripheral resistance TPR. Figure 3 shows the distribution of PP values computed from aortic, femoral, and radial ABP signal at each time point.

**TABLE 1:** Values of hemodynamic parameters, clinical data, estimated arterial compliance (AC), and total peripheral resistance (TPR) at each time point of the experiment.

	**T1**	**T2**	**T4**	**Delta T2-T1**	**Delta T4-T1**	**Delta T4-T2**
**Aortic**						
PP	29.9 (28.2,31.6)	31.1 (26.3,34.6)	51.5 (48,61.1)*	3.1 (−1.6,4.5)	22.8 (19.8,32.2)	19.7 (18.9,27.1)
SAP	84.1 (79.3,86.3)	65.6 (62.1,69.5)	94.9 (92.3,100.3)^§§^	−18.3 (−20.9,−16.3)	9.3 (6.2,14)	26 (24.3,32.3)
DAP	55.2 (50.5,56.6)	35.1 (33.6,35.8)*	45 (30,48.7)	−18.5 (−24.4,−15)	−11.9 (−18.3, −9)	7.5 (−1.2,12.9)
MAP	70.1 (65.9,71.8)	45.3 (45.2,45.5)*	67.6 (53.2,70.5)	−23.3 (−26.5,−18.9)	−3.5 (−12.9,0.6)	18.8 (7.8,23.9)
**Femoral**						
PP	44.9 (41.1,47.1)^##^	25.5 (22,26.2)*	34 (28.7,42.1)^#^	−19.2 (−24.3, −14.1)	−12.5 (−19.9,2.9)	7.4 (2.6,17)
SAP	102.4 (95.1,105.3)^##^	62.2 (55.5,63.5)**	82.4 (70.3,92.5)	−39.6 (−41.8,−36.5)	−22.5 (−30.4,−8)	14.9 (9.2,31.5)
DAP	57.5 (51.2,58.5)	36.6 (35.8,38.4)**	44.2 (40.7,49.7)	−19.8 (−21.1,−16.6)	−10.7 (−11.6,−8.5)	8.3 (4.8,14.4)
MAP	74.9 (68.7,75.8)	47.9 (45.4,49.8)**	64.4 (54.8,66.9)	−25.4 (−28,−23.3)	−9.1 (−16.4,−5.2)	14.9 (6.9,24.4)
**Radial**						
PP	46.8 (41.3,48.6)^##^	29.7 (22.8,34.4)	56.4 (46.8,63.3)^§^	−14.4 (−24.6,−13.8)	8.1 (−1.8,22.5)	27.8 (11.2,36.5)
SAP	100.6 (96.5,101.7)^##^	64 (60.8,68.6)*	97.4 (80.6,110.9)	−36.7 (−40.2,−34.1)	−5.5 (−15.9,11.2)	32 (12,47.3)
DAP	54.6 (51.5,55.3)	36.3 (33,38)**	41 (35,47.6)	−17.7 (−22,−14.9)	−13.7 (−14.1,−11.3)	4.4 (2.6,10.8)
MAP	71.6 (66.9,73)	45.6 (44.9,46.1)**	62.3 (52.8,68.9)	−25.4 (−27.8,−21.2)	−9.4 (−12.9,−4.3)	16 (6.7,23.5)
**HR**	82.1 (76.3,85.3)	131 (103,158.8)	146 (144,150)*	43.4 (23.9,78.4)	66.9 (64.9,68.8)	12 (−13.5,44)
**CO**	5 (4.5,5.3)	2.97 (2.4,4.3)^*n=5*^	9.5 (8.2,10.2)^§^	−1.9 (−2.2,−0.8)^*n=5*^	4.9 (2.8,6.3)	5.2 (4.4,6.3)^*n=5*^
**SV**	59 (52,60.5)	23.3 (20.1,36.8)^*n=5*^	65.5 (57.6,69.6)^§^	−29.5 (−35,−21.7)^*n=5*^	7.3 (−1.9,16.8)	34.3 (17,45)^*n=5*^
**UO**	1 (0.53,1.1)	0.1 (0.06,0.45)	0.22 (0.2,0.24)	−0.7 (−0.98,−0.2)	−0.8 (−0.9,−0.6)	0.1 (0,0.2)
**Lact**	0.9 (0.9,0.9)	1.7 (1.3,2.1)	2.45 (1.9,3.1)**	0.8 (0.4,1.2)	1.55 (1,2.2)	0.45 (0.1,1)
**SvO_2_**	65 (61,69)	59.5 (53,68)	75 (68,78)	−6.5 (−11,1)	9 (3,15)	14.5 (−1,23)
**PPV**	8 (7,10)	22.5 (17,30)**	11.5 (11,12)	14 (9,20)	4 (2,6)	−10 (−13,−5)
**AC** (SV/PP)	1.98 (1.8,2.14)	0.88 (0.7,1.05)^n=5^*	1.17 (1.05,1.34)	−1.19 (−1.36,−0.85)^*n=5*^	−0.78 (−0.99,−0.48)	0.38 (−0.03,0.57)^*n=5*^
**AC** (area)	1.96 (1.62,2.07)	0.9 (0.74,1.33)^n=5^*	1.11 (0.95,1.17)	−0.79 (−1.29,−0.54)^*n=5*^	−0.8 (−1.12,−0.4)	0.19 (−0.3,0.35)^*n=5*^
**TPR**	1124 (1061,1286)	1217 (879,1626)^*n=5*^	568 (437,676)^§^	155 (−161,363)^*n=5*^	−572 (−870,−420)	−540 (−918,−447)^*n=5*^

PP, pulse pressure (mmHg); MAP, mean pressure (mmHg); SAP, systolic pressure (mmHg); DAP, diastolic pressure (mmHg); HR, heart rate (bpm); CO, cardiac output (l/min); SV, stroke volume (ml); UO, urine output (l); Lact, lactate (mmol/l); SvO_2_, mixed venous oxygen saturation (%); PPV, pulse pressure variation (%); AC, total arterial compliance (ml/mmHg); TPR, total peripheral resistance (dyne^∗^s^∗^cm^–5^). T1, baseline; T2, after development of septic shock; T4, after resuscitation with fluids and noradrenaline. Wilcoxon signed test: *p < 0.05, **p < 0.01 with respect to T1, ^§^*p*-value < 0.05, ^§§^*p*-value < 0.01 with respect to T2 (Friedman test *p*-value < 0.05). Mann–Whitney *U*-test: ^#^*p* < 0.05, ^##^*p* < 0.01 with respect to aortic pressure at the specified time point. Values are reported as median (25th, 75th percentile).

At baseline (T1), PP was progressively higher from aortic to the more distal arterial sampling sites, reflecting the physiological peripheral PP amplification. The large increase in peripheral PP (∼15 mmHg) was primarily due to a higher value in SAP, since DAP values resulted similar at all sampling sites; MAP was not significantly different at the three sites. HR, CO, SV, and SvO_2_ have values within the physiological range. The arterial compliance AC was near the physiological value of 2 ml/mmHg (Mark, 2004).

After development of septic shock (T2), aortic PP slightly increased with no significant changes, whereas both femoral and radial PP progressively decreased and became slightly lower than aortic PP (Figure 3). The decrease in peripheral PP was due primarily to a larger decrease in SAP values in the peripheral sites as DAP values tended to decrease to a similar amount in all sites. HR increased as compensatory mechanism to sustain a condition of reduced venous return, CO, and SV (Table 1). The rise in lactate values and the moderate decrease in SvO_2_ denote the potential anaerobic cellular metabolism and heterogeneous microcirculatory alterations typical of shock. AC was more than halved in all pigs, hinting a certain degree of arterial stiffness.

**FIGURE 4:**
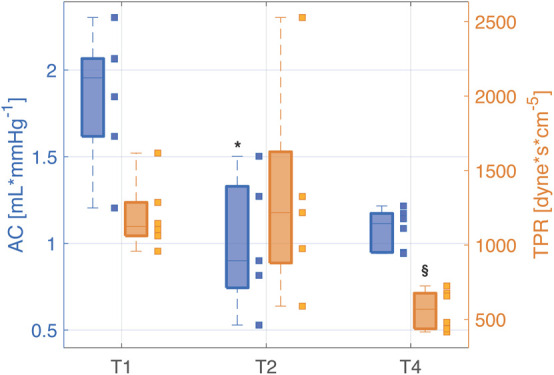
Distributions (median [25th, 75th percentile]) of total arterial compliance (AC) and total peripheral resistance (TPR) at each time point of the experiment. The values relating each pig are marked by the little squares. T1, baseline; T2, after development of septic shock; T4, after resuscitation with fluids and noradrenaline. Wilcoxon signed test: *p-value < 0.05 with respect to T1, ^§^ *p*-value < 0.05 with respect to T2 (Friedman test *p*-value < 0.05).

After full resuscitation with fluids and vasopressors (T4), the hypoperfusion and hypovolemic condition was restored as MAP increased to >60 mmHg, SV returned to values similar to baseline, SvO_2_ increased becoming higher than baseline values, and all the main hemodynamic indices, e.g., CO, SAP, DAP, showed a positive trend, i.e., the differences between the values at T4 and T2 were positive (Table 1). However, the arterial pressure variables at different sites did not return to the baseline values, in particular, the physiological PP amplification was not restored as in aorta PP values did not return to be significantly lower than in peripheral arteries. These differences in PP were driven by SAP, as DAP changed similarly at all sites. The SV returned to values comparable to baseline, but CO was almost doubled, as expected in the compensatory hyperdynamic phase of a resuscitated distributive shock, mainly due to a sustained tachycardia. This inefficient cardiac condition, despite a full resuscitation and the β1 adrenergic stimulation provided by the noradrenaline administration, indicates that the left ventricle was not able to provide a higher and adequate SV with the lowest possible energetic consumption, as already reported in a previous study of our group (Carrara et al., 2019). The values of AC were larger than in non-resuscitated shock but still lower than baseline. These results suggest that the large increase in central PP observed after resuscitation may be mostly due to the reduction in arterial compliance rather than an actual increase in heart efficiency as SV was only slightly increased compared to baseline (Table 1 and Figures 3, 4). Interestingly, a further rise in lactate was observed at T4. We may hypothesize that this could be rather due to the increase in aerobic glycolysis—induced by the adrenergic stimulation from the noradrenaline perfusion—with a saturation of the Pyruvate deshydrogenase enzyme leading to a shift toward lactate production. Indeed, recent findings support the evidence that serum lactate level should not be rigorously interpreted as an indication of hypoxia in septic shock. Moreover, numerous experimental data have demonstrated that stimulation of aerobic glycolysis occurs not only in resting, well-oxygenated skeletal muscles but also during experimental sepsis, and is closely linked to stimulation of sarcolemmal Na^+^/K^+^-ATPase under epinephrine stimulation (Levy, 2006). Finally, the evolution of the veno-arterial pCO_2_ gap during the experiment supports this argument that microcirculation derangements and cellular dysoxia were improved under noradrenaline perfusion, on top of fluid resuscitation, despite the small rise in lactate level at T4. Results of pCO_2_ VA-gap in mmHg (median [IQ range]) are: 7.8 [6.5–8.9] at baseline, 12 [10–15.5] at T2, and normalized at 4.7 [4.4–5.3] at T4.

It is important to recall that a decrease in ABP could alter arterial compliance, independently on the sepsis condition, due to the volume–pressure relationship (Westerhof et al., 2019). However, systemic hypotension, typical of circulatory shock state, would underfill the central vessels increasing arterial compliance. So the persisting decrease in AC in our experiment cannot be explained by the decrease in the arterial pressure. The same reasoning can also apply to the change in compliance observed from T2 to T4, where both AC and central blood pressure increased.

The estimated TPR showed a significant decrease at T4 with respect to un-resuscitated shock condition (T2) (Figure 4).

**FIGURE 5:**
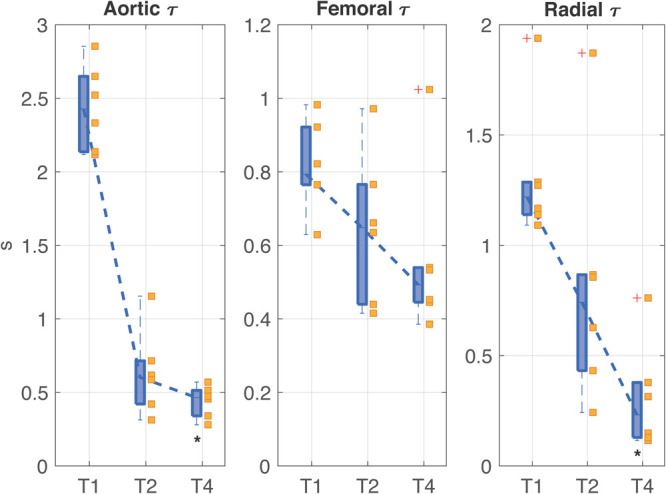
Distributions (median [25th, 75th percentile]) of the time constant τ values computed in aorta, femoral artery, and radial artery at each time point of the experiment. The values relating each pig are marked by the little squares. T1, baseline; T2, after development of septic shock; T4, after resuscitation with fluids and noradrenaline. Wilcoxon signed test: *p-value < 0.05 with respect to T1 (Friedman test *p*-value < 0.05).

Figure 5 shows the trend of the time constants τ computed at the three arterial sites (i.e., aorta, femoral artery, and radial artery) at each time point of the experiment. All the time constants were characterized by a monotonically decreasing trend from T1 to T4; statistical significance was reached at T4 with respect to T1 only in aortic and radial τ. The time constant τ is a global characteristic of the arterial tree, reflecting both the contribution of total arterial compliance and total peripheral resistance of the overall arterial circulation. However, one could notice a slightly different trend in the values of τ computed at aortic level and in the periphery; this may be partly explained by a different balance between elastic and resistive components present at central and peripheral arterial sites, with a predominance of decreased elastic properties in the aorta and a predominance of loss of peripheral resistance in femoral and radial arteries. Moreover, the trend of both AC and TPR (Figure 4 and Table 1) reinforces this assumption as in shock (T2) the main driver of the decreased time constant seems to be the reduction in arterial compliance, whereas at T4 a dramatic loss of resistance further contributes to the low values of τ.

Table 2 reports the values of the normalized LFu of DAP series. The index showed a monotonically decreasing trend from T1 to T4, hinting a persistent dysregulation of peripheral resistance by the ANS.

**TABLE 2:** LF normalized power computed for diastolic blood pressure components at each time point.

	**T1**	**T2**	**T4**	**Delta T2–T1**	**Delta T4–T1**	**Delta T4–T2**
**DAP LFu (%)**						
Aortic	7.9 (6.5,10.4)	2.2 (0.5,4)*	1.3 (1,2.9)	−5.5 (−8.9,−3.8)	−5 (−9.5,−3.5)	0.4 (−0.6,0.9)
Radial	9.3 (6.4,10.9)	2.3 (0.8,3.6)*	1.6 (1.3,3.8)	−7.6 (−7.9,−3.9)	−5.5 (−9,−3.4)	0.6 (−1.2,1.3)
Femoral	8.9 (8.2,11.9)	1.6 (0.7,3.9)*	1.7 (1.1,2.8)	−7.3 (−10.6,−5.1)	−6.2 (−10.7,−5.4)	0.6 (−0.2,1.5)

DAP, diastolic arterial pressures; LFu, normalized low frequency power. T1, baseline; T2, after development of septic shock; T4, after resuscitation with fluids and noradrenaline. Wilcoxon signed test: *p < 0.05 with respect to T1 (Friedman test *p*-value < 0.05). Values are reported as median (25th, 75th percentile).

Table 3 shows the results obtained from baroreflex sensitivity (BRS) analysis and HRV analysis. The FB gain, i.e., the autonomic-mediated baroreflex mechanism, was decreased during shock (T2) and was even further depressed after resuscitation (T4). A similar trend was observed also for the HRV indices. On the opposite, an increasing trend was observed for the FF gain, or runoff effect, which was higher compared to baseline both in shock and after resuscitation.

**TABLE 3:** Baroreflex feedback and feedforward gains and HRV indices computed at each time point.

	**T1**	**T2**	**T4**	**Delta T2–T1**	**Delta T4–T1**	**Delta T4–T2**
**BRS**						
FB (ms/mmHg)	1.7 (1.1,4.8)	0.3 (0.2,0.7)	0.2 (0.1,0.3)*	−1.4 (−4.5,−0.7)	−1.6 (−4.5,−1)	−0.2 (−0.4,0.1)
FF (mmHg/ms)	0.02 (0.02,0.08)	0.1 (0.04,0.3)	0.2 (0.19,0.21)	0.05 (0,0.3)	0.1 (0.08,0.2)	0.06 (−0.1,0.2)
**HRV**						
RMSSD (ms)	70.2 (49.9,86.6)	31.3 (25.9,56.8)	25.6 (22.5,29.4)*	−33.7 (−54,−15.9)	−45.1 (−60,−20.5)	−6.8 (−27.4,−3.6)
SDSD (ms)	3.7 (2.6,4.5)	1.6 (1.4,3)	1.3 (1.2,1.5)*	−1.8 (−2.8,−0.8)	−2.4 (−3.1,−1)	−0.4 (−1.4,−0.2)
SD (ms)	4.9 (3.5,7.6)	1.9 (1.6,4)	1.4 (1.2,1.8)*	−2.7 (−5.8,−0.5)	−3.5 (−6.2,−2.1)	−0.6 (−2.2,−0.4)

BRS, baroreflex sensitivity; FB, feedback gain; FF, feedforward gain; HRV, heart rate variability; RMSSD, root mean square of successive differences; SDSD, standard deviation of successive differences; SD, standard deviation. T1, baseline; T2, after development of septic shock; T4, after resuscitation with fluids and noradrenaline. Wilcoxon signed test: *p < 0.05 with respect to T1 (Friedman test *p*-value < 0.05). Values are reported as median (25th, 75th percentile).

**FIGURE 6:**
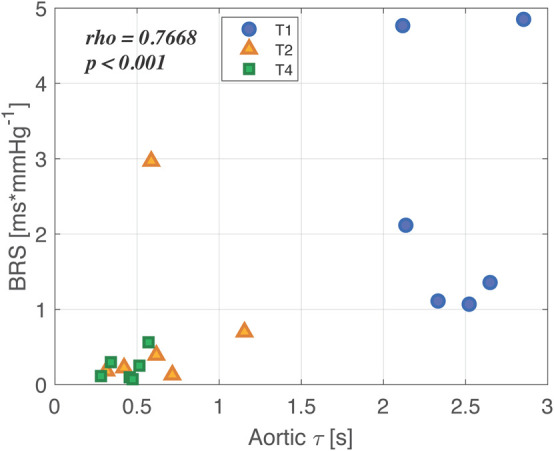
Correlation between characteristic time constant τ computed in aorta and baroreflex sensitivity (BRS) throughout the overall experiment. Baseline values (T1) are represented as blue circles, shock values (T2) as orange triangles, and values after full resuscitation with fluids and noradrenaline (T4) are reported as green small squares. In the upper left corner, the correlation coefficient and the *p*-value are reported.

Results of the correlation analysis are reported in Figure 6 for BRS and aortic τ (similar results, not shown, were found for the other variables). A strong positive correlation between the two indices was observed during the overall time course of the experiment. In particular, we noticed that the values at baseline were characterized both by an aortic τ higher than 2 s, representing the typical physiological value for humans (Mark, 2004), and by a BRS higher than 1 ms/mmHg. After shock and resuscitation, the values can be grouped in an area characterized by much lower values of both BRS and τ. This further highlights that after resuscitation either the vascular mechanics or the autonomic control did not return to the baseline condition.

## Discussion

In this study, a characterization of the arterial tree changes in mechanical properties, both at central and peripheral arterial sites, was investigated in a population of six pigs undergoing a fecal peritonitis-induced septic shock, including full resuscitation with fluids and noradrenaline. The interaction between the ANS dysfunction and these vascular changes were also assessed.

Our study confirms that septic shock induces a decoupling of the arterial pulse wave propagation from central to peripheral sites, without resolution after full resuscitation with fluids and noradrenaline. The ANS-related indices, i.e., indices of BRS, arterial pressure spectral analysis, and HRV, showed that septic shock produces signs of autonomic dysfunction and these are associated to changes in vascular properties, both at central and peripheral arterial sites.

### Vascular Changes During Shock and Following Resuscitation

An inversion of the physiological PP amplitude propagation from central to peripheral arteries was observed after the development of septic shock, as already reported in a recent study of endotoxin shock (Hatib et al., 2011). Of interest, in this study, we also observed a persistent vascular decoupling condition hours after a full resuscitation with fluids and noradrenaline (Table 1 and Figure 3). This finding, together with the trend of AC, TPR, and the Windkessel time constants, hints an altered behavior of the vascular compartments induced by septic shock.

The origin of this PP reversal could be related to the different tissue composition of central and peripheral arterial walls and, thus, to a different response to inflammatory vasoactive substances, such as nitric oxide (NO).

In large compliant arteries, local endogenous NO generation has been demonstrated to contribute to the regulation of large-artery stiffness at basal condition (Wilkinson et al., 2002), and it was observed that acute systemic inflammation impairs the ability of the arterial endothelium to produce endogenous vasodilators in response to agonist and physical stimuli (Hingorani et al., 2000). Reduced endogenous NO availability contributes to endothelial dysfunction during sepsis and acute inflammation, and it may lead to a functional stiffening of the large arteries (Clapp et al., 2004).

In peripheral resistance vessels, NO is recognized to be crucial in the modulation of vasomotor tone, and a growing body of evidence indicates that NO plays an important role in the hyporesponsiveness of resistance vessels to vasoactive agents (Lush and Kvietys, 2000). The cellular source of NO generation within the microvasculature during sepsis is not completely clear, but there is some evidence to indicate that vascular smooth muscle, mostly present in peripheral vessels, may be the source (Lush and Kvietys, 2000). In septic shock patients, plasma concentrations of NO metabolites are markedly increased, and it was found that the raised production of NO is the result of the increased expression of the inducible form of NO synthase (iNOS), probably stimulated by the inflammatory cytokines (Landry and Oliver, 2001). This induces a persistent vasodilation despite high plasma concentrations of catecholamines (Young, 2004).

The differences in structural components between large complaint and peripheral resistance vessels could partly explain the different endothelial response to inflammation: large-artery stiffening is caused by a reduction in NO bioavailability following inflammation and sepsis, and, conversely, the persistent peripheral vasodilation is caused by the large increase in NO at peripheral level occurring in sepsis and shock.

Although NO bioavailability was not measured in our experiment, in a previous work (Ferrario et al., 2019), we observed a decrease of the global arginine bioavailability, i.e., the ratio arginine/(ornithine+citrulline), in the same septic shock animals. As arginine is the precursor for the biosynthesis of NO, our data are consistent with the hypothesis of an increase in NO production supporting the previously outlined mechanisms.

Another important factor that can contribute to PP reversal and peripheral vascular decoupling observed in these animals may be a modification in the reflection of pressure waves. In Vlachopoulos et al. (2005), the authors show that acute systemic inflammation in healthy individuals increases arterial stiffness, assessed by PWV, and decreases wave reflection, estimated by means of the augmentation index on central aortic pressure waveform. The authors explained this phenomenon by the predominant mechanism of peripheral vasodilation. In our study, the estimated TPR and AC decrease in agreement with this work.

Finally, the increased capillary permeability secondary to endothelial dysfunction may also contribute to the general vascular derangements observed in these animals. High capillary leakage may lead to changes in extracellular matrix composition and this, in turn, could have an influence on the contraction and relaxation properties of the vessels.

The vascular dysfunction induced by septic shock could also help in explaining the increase in FF gain, which measures the direct influence of heart interval duration on SAP, not mediated by autonomic control, but instead by a perturbation mechanism based on the Starling law and diastolic runoff (Table 3). The decreased arterial compliance inevitably limits the Windkessel effect of the large arteries (Westerhof et al., 2009), and, as a consequence, the blood flow could arrive at peripheral districts with a higher pulsatility; this, combined with the vasoplegic state typical of septic shock, could result in amplified oscillations in ABP induced by an elevated HR.

### Autonomic Dysfunction and Association With Vascular Changes

The autonomic indices of cardiovascular regulation such as BRS, HRV, and LF normalized power of DAP all showed a depressed value in shock which persisted, or even worsened, after full resuscitation (Tables 2, 3). Thus, a condition of autonomic dysfunction, not resolved by the resuscitation maneuvers, is clearly depicted in these animals.

On the basis of our data, we cannot say if this autonomic impairment involves the central or peripheral nervous system or both. Sepsis-associated encephalopathy is commonly observed in septic patients, and, although its pathophysiology has not been established yet, several processes are known to contribute to its generation, such as brain ischemia, inflammation, metabolic and mitochondrial dysfunction, altered cerebral blood flow autoregulation, or blood–brain barrier leakage (Gofton and Bryan Young, 2012). A central impairment could help to explain our result of a depressed value of ANS indices which persisted also after resuscitation; however, in a previous study of our group, we found that the autonomic-mediated baroreflex control of ventricular contractility was enhanced both during septic shock and after full resuscitation in the same experimental pig population, on the opposite of the observed depressed HRV (Carrara et al., 2019). Other literature works also report a differentiated stimulation of peripheral ANS, with enhanced autonomic activity at some districts and depressed at others (Vayssettes-Courchay et al., 2005; Ramchandra et al., 2009). Based on these findings, we cannot state that a dysfunction of the central nervous system may exhaustively explain the ANS impairment observed in these animals. Other mechanisms may also play a role, for example, disturbances in the efferent or afferent nerve fibers, downregulation and hyporesponsiveness of target receptors, or a dysfunction of the baroreflex system. The overwhelming inflammation may likely trigger, at least in part, these complications. For example, a reduced sensitivity of the aortic baroreceptors caused by the increased stiffness of the vessel may contribute to the baroreflex failure (Bonyhay et al., 1996).

Typically, a reduced baroreflex FB gain and a depressed HRV, together with an elevated HR, are interpreted as signs of a depressed vagal activity at the heart level. These observations suggest a vagal driver for an effective resuscitation, and this hypothesis is in line with the recent literature on vagus nerve stimulation as an innovative therapeutic strategy, which has been proven to be beneficial also in septic shock (Borovikova et al., 2000; Tracey, 2002; Liu et al., 2006; Bansal et al., 2010; Czura et al., 2010; Huston and Fritz, 2018).

The depressed LFu power of DAP hints a dysfunction of the sympathetic nervous system also in the periphery. Changes in LF oscillations of arterial pressure can be related to changes in the outflow of the sympathetic nervous system, and spectral analysis of arterial pressure has been proven to be a powerful tool for identification of the different cardiovascular control mechanisms that regulate arterial pressure (Stauss et al., 1998; Stauss, 2007). From this perspective, the reduction of LF power can be interpreted as a withdrawal or a saturation of sympathetic activity, or an inability of adrenergic receptors to respond to further stimuli.

There are many recent indirect and direct evidences which suggest a very complex link between sympathetic activity and vascular function. Evidence from experimental studies indicates that the sympathetic ANS is influenced, both at central and peripheral level, by the most relevant factors regulating vascular function (Bruno et al., 2012). For example, neuronal NOS (nNOS) is constitutively expressed in neuronal cells of both peripheral and central nervous system, and in the latter it acts as a sympathoinhibitory substance (Patel et al., 2001). Moreover, a relationship between arterial stiffness and sympathetic nerve traffic was also demonstrated, with the majority of studies reporting a positive correlation between sympathetic withdrawal and increased large artery elasticity (Skrapari et al., 2007; Liatis et al., 2011; Bruno et al., 2012; Theilade et al., 2013; Millar et al., 2019). In our study, we can also confirm this association, as our analyses highlighted a significant positive correlation between a decrease in arterial compliance, i.e., increase in large artery stiffness, and a depressed vagal activity, as shown by the suppressed BRS, HRV, and LFu index.

The relationship between autonomic and vascular dysfunction could be causal in both directions, i.e., autonomic impairment generates dysfunction in vascular tone or vice versa, or non-causal, i.e., the two conditions may develop in parallel as inflammatory responses. Sympatho-excitatory maneuvers were found to impair endothelial function and to increase arterial stiffness, and markers of vascular dysfunction were shown to be inversely related to sympathetic discharge (Bruno et al., 2012). On the other hand, large artery stiffness can interfere with autonomic regulation by impairing arterial baroreceptors, as already discussed above. All these observations support a causality mechanism.

Another causal mechanism could be the increase in HR, as a sympathetic-mediated increase in HR per se results in stiffening of the central arteries (Liatis et al., 2011). Finally, arterial elasticity could modify autonomic activity through a change in cardiac afterload. An increase in arterial stiffness leads to an increased afterload, such that the heart needs a greater effort to eject the same SV, with a consequent sympathetic overstimulation to increase HR and ventricular contractility; if prolonged, this condition could lead to heart failure, considering also that an increased HR results in a shorter time for diastole. Moreover, an increased stiffness leads to an increase in PWV along the arterial tree with the consequence that the reflected waves arrive at the heart earlier in systole further increasing the cardiac afterload. These conclusions were also recently pointed out by Monge García et al. (2018), who showed that high doses of noradrenaline administration in septic shock patients increased arterial characteristic impedance, PWV and reflection phenomena, and reduced aortic compliance. Therefore, even if MAP is restored with hemodynamic resuscitation, these therapeutic interventions may have a deleterious effect on the efficiency of the energy transfer from the ventricle to the periphery. This phenomenon could partly explain the persistent tachycardia and the elevated contractility observed in these animal population (Carrara et al., 2019) and generally reported in septic shock patients (Merx and Weber, 2007; Rudiger and Singer, 2013; Morelli et al., 2015; Beesley et al., 2017).

**FIGURE 7:**
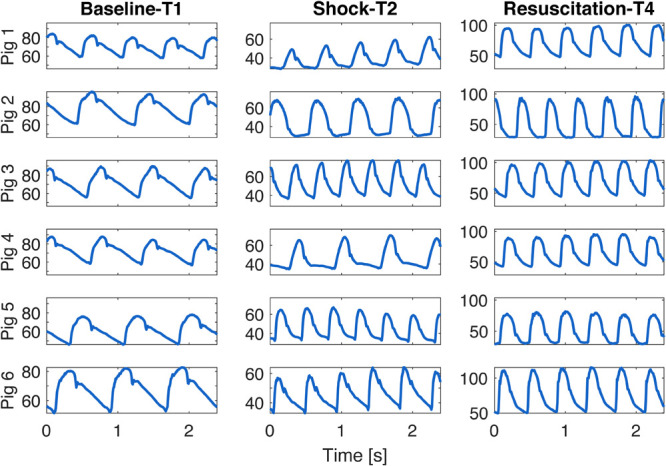
Example of few beats extracted from aortic waveform at each time point of the experiment for each animal.

In conclusion, although this study did not demonstrate a direct causal relationship between vascular dysfunction and ANS activity, our observations clearly proved that both the vascular and the autonomic system are impaired in septic shock and they mutually influence each other. This compromised condition is not resolved by standard therapy, i.e., fluids and noradrenaline administration, and it can be macroscopically appreciated from the changes in morphology of blood pressure waveforms. Few cardiac beats of aortic pressure waveform are shown in Figure 7 for all the animals at each time point of the experiment. Interestingly, the shape of the aortic waveform mostly at T4 resembles the typical pressure waveform of a high cardiovascular risk subject, such as aged people as reported in Izzo (2007), although these changes were developed only in a few hours of experiment.

### Limitations and Future Developments

The main limitation of this study consists in the small sample size, which may be the source of a high variability in the results so to affect the statistical tests. However, the results showed evident trends that can still be considered important, even if not statistically significant. Further studies with a larger number of subjects will permit to validate the results herein presented.

Moreover, direct measures of peripheral arteries compliance and autonomic outflow were not available and this did not permit other than speculation about possible associations. Also, the lack of an invasive local flow measurement at some of the arterial pressure measurement sites impeded us to compute the local characteristic impedance, but it was the price to pay in order to keep a closed chest, closed pericardium model, with intact cardiopulmonary and interventricular interactions and less tissue damages.

Finally, it could be worthy to integrate these analyses with molecular mechanism involved by shock and resuscitation, since vasoactive agents released during sepsis, such as NO, have a directly influence on the hemodynamic response to shock, and the responsible mechanisms still remain to be elucidated.

## Conclusion

The present study proposed an insightful analysis of the changes of vascular properties occurring during a protocol of polymicrobial septic shock and full resuscitation, and suggested a possible relationship with autonomic dysfunction typical of septic shock. In fact, a significant positive correlation between an increase in arterial stiffness and a depressed vagal activity has been reported, together with a decreased TPR and time constant τ and an impaired sympathetic activity at the peripheral sites. Interestingly, this abnormal condition generated by septic shock was not resolved after administration of fluids and noradrenaline. Although global hemodynamic markers were restored by the resuscitation maneuvers, such as MAP, HR, CO, and SV, as shown in Table 1, we observed that indices of cardiovascular functions mediated by the ANS were still impaired, suggesting that the homeostatic condition of baseline was not completely recovered, as already reported in our previous studies (Carrara et al., 2018a, b, 2019).

The discussed results foster further investigations on the integrative aspects of septic shock-induced cardiovascular dysfunction, and they may suggest that the current resuscitation strategies could benefit from the adjunction of additional functional indices combined to the standard hemodynamic and clinical markers. Moreover, our study suggests that measures of vascular properties should be taken into account in further studies in order to have a more comprehensive picture of the effectiveness of the therapy. The computation of time constant τ is a powerful approach to investigate the vascular behavior in terms of total compliance and resistance, and it could be easily computed by the recorded pressure waveform.

Finally, a more effective vasopressor therapy should take into account the different characteristics between central and peripheral arteries and further studies should be performed to understand the right balance in order to restore not only MAP but also a physiological condition of cardiovascular interactions and arterial pressure control.

## Data Availability Statement

The datasets generated for this study are available on request to the corresponding author.

## Ethics Statement

The animal study was reviewed and approved by Comité Ethique du Bien-Être Animal, Experimental Laboratory of Intensive Care (LA1230336), Université Libre de Bruxelles.

## Author Contributions

MF, AH, and GB conceived and designed the research. AH performed the experiments. MC analyzed the data, prepared figures, and drafted the manuscript. MC, MF, and AH interpreted the results and edited and revised the manuscript. MC, MF, AH, GB approved the final version of the manuscript. All authors contributed to the article and approved the submitted version.

## Conflict of Interest

The authors declare that the research was conducted in the absence of any commercial or financial relationships that could be construed as a potential conflict of interest.

## References

[B1] Bansal V. Costantini T. Ryu S. Peterson C. Loomis W. Putnam J. (2010). Stimulating the central nervous system to prevent intestinal dysfunction after traumatic brain injury. *J. Trauma.* 68 1059–1064. 10.2217/nnm.12.167.Gene 20453760 PMC4251579

[B2] Barbieri R. Parati G. Saul J. P. (2001). Closed- versus open-loop assessment of heart rate baroreflex. *IEEE Eng. Med. Biol. Mag.* 20 33–42. 10.1109/51.917722 11321718

[B3] Beesley S. Wilson E. Lanspa M. Grissom C. Shahul S. Talmor D. (2017). Persistent tachycardia and mortality in septic shock patients. *Am. J. Respir. Crit. Care Med.* 195:A1899. 10.1097/CCM.0000000000002065 PMC5512273 27618277

[B4] Bonyhay I. Jokkel G. Kollai M. (1996). Relation between baroreflex sensitivity and carotid artery elasticity in healthy humans. *Am. J. Physiol. Hear. Circ. Physiol.* 271 H1139–H1144. 10.1152/ajpheart.1996.271.3.H1139 8853352

[B5] Borovikova L. V. Ivanova S. Zhang M. H. Yang H. Botchkina G. I. Watkins L. R. (2000). Vagus nerve stimulation attenuates the systemic inflammatory response to endotoxin. *Nature* 405 458–462. 10.1038/35013070 10839541

[B6] Bourgeois M. J. Gilbert B. K. Donald D. E. Wood E. H. (1974). Characteristics of aortic diastolic pressure decay with application to the continuous monitoring of changes in peripheral vascular resistance. *Circ. Res.* 35 56–66. 10.1161/01.RES.35.1.56 4366522

[B7] Bruno R. M. Ghiadoni L. Seravalle G. Dell’Oro R. Taddei S. Grassi G. (2012). Sympathetic regulation of vascular function in health and disease. *Front. Physiol.* 3:284. 10.3389/fphys.2012.00284 22934037 PMC3429057

[B8] Carrara M. Babini G. Baselli G. Ristagno G. Pastorelli R. Brunelli L. (2018a). Blood pressure variability, heart functionality and left ventricular tissue alterations in a protocol of severe hemorrhagic shock and resuscitation. *J. Appl. Physiol.* 125 1011–1020. 10.1152/japplphysiol.00348.2018 30001154 PMC6230573

[B9] Carrara M. Bollen Pinto B. Baselli G. Bendjelid K. Ferrario M. (2018b). Baroreflex sensitivity and blood pressure variability can help in understanding the different response to therapy during acute phase of septic shock. *Shock* 50 78–86. 10.1097/SHK.0000000000001046 29112634 PMC5991174

[B10] Carrara M. Herpain A. Baselli G. Ferrario M. (2019). A mathematical model of dP/dt max for the evaluation of the dynamic control of heart contractility in septic shock. *IEEE Trans. Biomed. Eng.* 66 2719–2727. 10.1109/tbme.2019.2894333 30872214

[B11] Carthy E. R. (2014). Autonomic dysfunction in essential hypertension: a systematic review. *Ann. Med. Surg.* 3 2–7. 10.1016/j.amsu.2013.11.002 25568776 PMC4268473

[B12] Clapp B. R. Hingorani A. D. Kharbanda R. K. Mohamed-Ali V. Stephens J. W. Vallance P. (2004). Inflammation-induced endothelial dysfunction involves reduced nitric oxide bioavailability and increased oxidant stress. *Cardiovasc. Res.* 64 172–178. 10.1016/j.cardiores.2004.06.020 15364625

[B13] Czura C. J. Schultz A. Kaipel M. Khadem A. Jared M. Pavlov V. A. (2010). Vagus nerve stimulation regulates hemostasis in swine. *Shock* 33 608–613. 10.1097/SHK.0b013e3181cc0183.VAGUS 19953009 PMC2921076

[B14] Ferrario M. Brunelli L. Su F. Herpain A. (2019). The systemic alterations of lipids, alanine-glucose cycle and inter-organ amino acid metabolism in swine model confirms the role of liver in early phase of septic shock. *Front. Physiol.* 10:11. 10.3389/fphys.2019.00011 30745875 PMC6360162

[B15] Gofton T. E. Bryan Young G. (2012). Sepsis-associated encephalopathy. *Nat. Rev. Neurol.* 8 557–566. 10.1038/nrneurol.2012.183 22986430

[B16] Granger C. W. J. (1963). Economic processes involving feedback. *Inf. Control* 6 28–48. 10.1016/s0019-9958(63)90092-5

[B17] Hatib F. Jansen J. R. C. Pinsky M. R. (2011). Peripheral vascular decoupling in porcine endotoxic shock. *J. Appl. Physiol.* 111 853–860. 10.1152/japplphysiol.00066.2011 21700890 PMC3174791

[B18] Hingorani A. D. Cross J. Kharbanda R. K. Mullen M. J. Taylor M. Donald A. E. (2000). Acute systemic inflammation impairs endothelium-dependent dilatation in humans. *Circulation* 102 994–999. 10.1161/01.cir.102.9.994 10961963

[B19] Huston J. M. Fritz J. R. (2018). The inflammatory reflex and neural tourniquet: harnessing the healing power of the vagus nerve. *Bioelectron. Med.* 1 29–38. 10.2217/bem-2017-0002

[B20] Ince C. Mayeux P. R. Nguyen T. Gomez H. Kellum J. A. Ospina-Tascón G. A. (2016). The endothelium in sepsis. *Shock* 45 259–270. 10.1097/SHK.0000000000000473 26871664 PMC5281063

[B21] Izzo J. L. (2007). Aging, arterial stiffness, and systolic hypertension. *Hypertens. Elder* 65 23–34. 10.1007/978-1-59259-911-0_3

[B22] Kazune S. Grabovskis A. Cescon C. Strike E. Vanags I. (2019). Association between increased arterial stiffness and clinical outcomes in patients with early sepsis: a prospective observational cohort study. *Intensive Care Med. Exp.* 7:26. 10.1186/s40635-019-0252-3 31098834 PMC6522594

[B23] Landry D. Oliver J. (2001). The pathogenesis of vasodilatory shock. *N. Engl. J. Med.* 345 588–595. 10.1056/nejmra002709 11529214

[B24] Leclerc J. Pu Q. Corseaux D. Haddad E. Decoene C. Bordet R. (2000). A single endotoxin injection in the rabbit causes prolonged blood vessel dysfunction and a procoagulant state. *Crit. Care Med.* 28 3672–3678. 10.1097/00003246-200011000-00023 11098972

[B25] Levy B. (2006). Lactate and shock state: a metabolic review. *Curr. Opin. Crit. Care* 12 315–321. 10.1097/01.ccx.0000235208.77450.15 16810041

[B26] Liatis S. Alexiadou K. Tsiakou A. Makrilakis K. Katsilambros N. Tentolouris N. (2011). Cardiac autonomic function correlates with arterial stiffness in the early stage of type 1 diabetes. *Exp. Diabetes Res.* 2011 1–7. 10.1155/2011/957901 21804819 PMC3143454

[B27] Liu C. Y. Mueller M. H. Grundy D. Kreis M. E. (2006). Vagal modulation of intestinal afferent sensitivity to systemic LPS in the rat. *AJP Gastrointest. Liver Physiol.* 292 G1213–G1220. 10.1152/ajpgi.00267.2006 17204546

[B28] Liu Z. Brin K. P. Yin F. C. P. (1986). Estimation of total arterial compliance: An improved method and evaluation of current methods. *Am. J. Physiol. Hear. Circ. Physiol.* 251(3 Pt 2), H588–H600. 10.1152/ajpheart.1986.251.3.H588 3752271

[B29] Lush C. W. Kvietys P. R. (2000). Microvascular dysfunction in sepsis. *Microcirculation* 7 83–101. 10.1038/sj.mn.7300096 10802851

[B30] Mark R. G. (2004). *Costanzo, Linda S. Physiology*. 2nd Edn. Philadelphia, PA: W.B. Saunders.

[B31] Merx M. Weber C. (2007). Sepsis and the heart. *Circulation* 116 793–802. 10.1093/bja/aep339 17698745

[B32] Millar P. J. Notarius C. F. Haruki N. Floras J. S. (2019). Heart failure–specific relationship between muscle sympathetic nerve activity and aortic wave reflection. *J. Card. Fail.* 25 404–408. 10.1016/j.cardfail.2019.03.005 30862489

[B33] Monge García M. I. Santos A. Diez Del Corral B. Guijo González P. Gracia Romero M. Gil Cano A. (2018). Noradrenaline modifies arterial reflection phenomena and left ventricular efficiency in septic shock patients: a prospective observational study. *J. Crit. Care* 47 280–286. 10.1016/j.jcrc.2018.07.027 30096635

[B34] Morelli A. Egidio A. D. Passariello M. (2015). “Tachycardia in septic shock: pathophysiological implications and pharmacological treatment,” in *Annual Update in Intensive Care and Emergency Medicine*, ed. Vincent J. L. (Cham: Springer), 10.1007/978-3-319-13761-2

[B35] Mukkamala R. Reisner A. T. Hojman H. M. Mark R. G. Cohen R. J. (2006). Continuous cardiac output monitoring by peripheral blood pressure waveform analysis. *IEEE Trans. Biomed. Eng.* 53 459–467. 10.1152/japplphysiol.00951.2006 16532772

[B36] Osuchowski M. F. Ayala A. Bahrami S. Bauer M. Boros M. Cavaillon J.-M. (2018). Minimum quality threshold in pre-clinical sepsis studies (MQTiPSS): an international expert consensus initiative for improvement of animal modeling in sepsis. *Shock* 50, 377–380. 10.1097/SHK.0000000000001212 30106875 PMC6133201

[B37] Patel K. P. Li Y. F. Hirooka Y. (2001). Role of nitric oxide in central sympathetic outflow. *Exp. Biol. Med.* 226 814–824. 10.1177/153537020122600902 11568303

[B38] Ramchandra R. Wan L. Hood S. G. Frithiof R. Bellomo R. May C. N. (2009). Septic shock induces distinct changes in sympathetic nerve activity to the heart and kidney in conscious sheep. *Am. J. Physiol. Regul. Integr. Comp. Physiol.* 297 R1247–R1253. 10.1152/ajpregu.00437.2009 19726712

[B39] Rhodes A. Evans L. E. Alhazzani W. Levy M. M. Antonelli M. Ferrer R. (2017). Surviving sepsis campaign: international guidelines for management of sepsis and septic shock: 2016. *Crit. Care Med.* 45 486–552. 10.1097/CCM.0000000000002255 28098591

[B40] Rudiger A. Singer M. (2013). The heart in sepsis: from basic mechanisms to clinical management. *Curr. Vasc. Pharmacol.* 11 187–195. 10.2174/1570161111311020008 23506497

[B41] Secrest A. Marshall S. Miller R. Prince C. Orchard T. (2011). Pulse wave analysis and cardiac autonomic neuropathy in type 1 diabetes: a report from the pittsburgh epidemiology of diabetes complications study. *Diabetes Technol. Ther.* 13 1264–1268. 10.1089/dia.2011.0126 21819228 PMC3263305

[B42] Skrapari I. Tentolouris N. Perrea D. Bakoyiannis C. Papazafiropoulou A. Katsilambros N. (2007). Baroreflex sensitivity in obesity: relationship with cardiac autonomic nervous system activity. *Obesity* 15 1685–1693. 10.1038/oby.2007.201 17636086

[B43] Stauss H. M. (2007). Identification of blood pressure control mechanisms by power spectral analysis. *Clin. Exp. Pharmacol. Physiol.* 34 362–368. 10.1111/j.1440-1681.2007.04588.x 17324151

[B44] Stauss H. M. Anderson E. A. Haynes W. G. Kregel K. C. (1998). Frequency response characteristics of sympathetically mediated vasomotor waves in humans. *Am. J. Physiol.* 274 H1277—-83. 9575932 10.1152/ajpheart.1998.274.4.H1277

[B45] Sun J. X. Reisner A. T. Mark R. G. G. Zong W. Moody G. B. Mark R. G. G. (2004). A signal abnormality index for arterial blood pressure waveforms. *Med. Biol. Eng. Comput.* 42 698–706. 15503972 10.1007/BF02347553

[B46] Swierblewska E. Hering D. Kara T. Kunicka K. Kruszewski P. Bieniaszewski L. (2010). An independent relationship between muscle sympathetic nerve activity and pulse wave velocity in normal humans. *J. Hypertens.* 28 979–984. 10.1097/hjh.0b013e328336ed9a 20408258

[B47] Task Force of the European Society of Cardiology and the North American Society of Pacing and Electrophysiology (1996). Heart rate variability. Standards of measurements, physiological interpretation, and clinical use. *Eur. Heart J.* 17 354–381. 10.1161/01.CIR.93.5.1043 8737210

[B48] Theilade S. Lajer M. Persson F. Joergensen C. Rossing P. (2013). Arterial stiffness is associated with cardiovascular, renal, retinal, and autonomic disease in type 1 diabetes. *Diabetes Care* 36 715–721. 10.2337/dc12-0850 23193205 PMC3579374

[B49] Tracey K. (2002). The inflammatory reflex. *Nature* 420 853–859. 10.1038/nature01321 12490958

[B50] Vayssettes-Courchay C. Bouysset F. Verbeuren T. J. (2005). Sympathetic activation and tachycardia in lipopolysaccharide treated rats are temporally correlated and unrelated to the baroreflex. *Auton. Neurosci. Basic Clin.* 120 35–45. 10.1016/j.autneu.2005.03.002 15996623

[B51] Vlachopoulos C. Dima I. Aznaouridis K. Vasiliadou C. Ioakeimidis N. Aggeli C. (2005). Acute systemic inflammation increases arterial stiffness and decreases wave reflections in healthy individuals. *Circulation* 112 2193–2200. 10.1161/CIRCULATIONAHA.105.535435 16186422

[B52] Wessel N. Voss A. Malberg H. Ziehmann C. Voss H. U. Schirdewan A. (2000). Nonlinear analysis of complex phenomena in cardiological data. *Herzschrittmachertherapie und Elektrophysiologie* 11 159–173. 10.1007/s003990070035

[B53] Westerhof N. Lankhaar J. Westerhof B. (2009). The arterial windkessel. *Med. Biol. Eng. Comput.* 47 131–141. 18543011 10.1007/s11517-008-0359-2

[B54] Westerhof N. Stergiopulos N. Noble M. I. M. Westerhof B. E. (2019). *Snapshots of Hemodynamics: An Aid for Clinical Research and Graduate Education.* Berlin: Springer.

[B55] Wilkinson I. B. Qasem A. McEniery C. M. Webb D. J. Avolio A. P. Cockcroft J. R. (2002). Nitric oxide regulates local arterial distensibility in vivo. *Circulation* 105 213–217. 10.1161/hc0202.101970 11790703

[B56] Young J. D. (2004). The heart and circulation in severe sepsis. *Br. J. Anaesth.* 93 114–120. 10.1093/bja/aeh171 15121730

[B57] Zong W. Heldt T. Moody G. B. Mark R. G. (2003). An open-source algorithm to detect onset of arterial blood pressure pulses. *Comput. Cardiol.* 2003 259–262. 10.1109/CIC.2003.1291140

